# Task shifting from general practitioners to practice assistants and nurses in primary care: a cross-sectional survey in 34 countries – RETRACTED

**DOI:** 10.1017/S1463423622000202

**Published:** 2022-05-17

**Authors:** Peter P. Groenewegen, Wienke G. W. Boerma, Peter Spreeuwenberg, Bohumil Seifert, Willemijn Schäfer, Ronald Batenburg, Lilian van Tuyl

We temporary retreat the paper by Groenewegen et al and will provide a revised version. The reason is that we have made a serious mistake in recoding the dependent variables that form the task shifting scale. Instead of recoding ‘not applicable (no nurse in my practice)’ into ‘no’, as stated in the method section, we have recoded it by mistake into ‘yes’. As a consequence Figure 1 and Table 1 are incorrect. The tables presented in the Supplementary Material are correct. The analysis has to be done anew and the conclusions will be partly different. For those countries that have a low number of practices with ‘not applicable (no nurse in my practice)’ the differences will be small and this is the majority of countries. However, the results and discussion sections have to be rewritten.

The mistake was made by the first author (PG) and the statistician (PS). They take full responsibility. The paper had undergone the normal procedures of checks by the other authors, the internal peer review within Nivel (according to their quality system) and external peer review by the journal. The mistake was found after a question by a reader who noted an inconsistency between Figure 1 and the information in Supplementary Tables 2-5. We thank her for pointing this out to us. We apologise to readers and to the journal.

The article PDF has been watermarked and the abstract and HTML version have been amended to state that the article has been retracted.
